# Systemic frequencies of T helper 1 and T helper 17 cells in patients with age-related macular degeneration: A case-control study

**DOI:** 10.1038/s41598-017-00741-4

**Published:** 2017-04-04

**Authors:** Amardeep Singh, Yousif Subhi, Marie Krogh Nielsen, Mads Krüger Falk, Sara Maj Hyldig Matzen, Finn Sellebjerg, Torben Lykke Sørensen

**Affiliations:** 1 Clinical Eye Research Unit, Department of Ophthalmology, Zealand University Hospital, Roskilde, Denmark; 2 Department of Ophthalmology, Skåne University Hospital Malmö-Lund, Lund, Sweden; 3 Faculty of Health and Medical Sciences, University of Copenhagen, Copenhagen, Denmark; 4 Department of Clinical Biochemistry, Zealand University Hospital, Roskilde, Denmark; 5 Danish Multiple Sclerosis Center, Department of Neurology, National University Hospital Rigshospitalet, Copenhagen, Denmark

## Abstract

Age-related macular degeneration (AMD) is a degenerative disease of the retina and a leading cause of irreversible vision loss. We investigated the systemic differences in the frequency of T helper (Th) 1 and Th17 cells in patients with non-exudative and exudative AMD and compared to age-matched controls. Flow cytometry was used to determine the systemic frequency of Th1 (CD4^+^CXCR3^+^IL12RB2^+^) and Th17 (CD4^+^CCR6^+^IL23R^+^) cells, and percentage of CD4^+^ T-cells expressing CXCR3, IL12RB2, CCR6, IL23R, and co-expressing CXCR3 and CCR6. The frequency of Th1 cells and CXCR3^+^ CD4^+^ T-cells was lower in patients with exudative AMD. A significant age-dependent decrement in Th1 was observed in controls, but not in non-exudative or exudative AMD. This may be related to the CXCR3^+^ CD4^+^ T-cells, which showed similar pattern in controls, but not in non-exudative or exudative AMD. No significant group differences were observed for the frequency of Th17 cells. Correlation networks found several differences between controls and AMD. These data suggests the involvement of the adaptive immune system in AMD and supports the notion of AMD as a systemic disease. Our observations warrant further investigation into the role of the adaptive immune system in the pathogenesis of AMD.

## Introduction

Age-related macular degeneration (AMD) is a degenerative disease of the photoreceptor cells, retinal pigment epithelium (RPE), and Bruch’s membrane in the macular region of the retina^1^. It is the most common cause of irreversible vision loss in the elderly and significantly impacts quality of life^2–4^. Early stages are usually asymptomatic and characterized by the presence of drusen, which are extracellular accumulations in proximity to the RPE. In the later stages, atrophy of the outer retina leads to gradual and irreversible vision loss. Rapid visual loss often ensues when choroidal vessels proliferate and break through the Bruch’s membrane to enter the sub-RPE or the subretinal space resulting in hemorrhage, exudation, or edema in the macula. This stage is commonly referred to as the neovascular or exudative AMD.

Despite recent advancements in the understanding of the pathogenesis of AMD, the underlying mechanisms are still incompletely understood. The immune system is believed to play an important role and it has been proposed that an imbalance between tissue stress (e.g. due to oxidative changes) and para-inflammatory responses (e.g. due to genetic mutations) is central to the development of AMD^5, 6^. Although AMD exclusively affects the posterior pole of the eye, immunological changes in the systemic circulation have been reported in patients with AMD, strongly indicating a systemic component in AMD^7–15^.

In understanding the pathogenesis of AMD, the attention has primarily been directed towards the innate immune system. However, there is some evidence to suggest that the adaptive immune system may also be involved to some degree. For instance, autoantibodies have been found in blood of patients with AMD^16, 17^ and CD8^+^ T-cells have been identified in the choroid of donated human eyes affected by AMD^18, 19^. Further evidence for involvement of the adaptive immune system arises from the observation that major histocompatibility complex class (MHC) II molecules are present on RPE and enhanced on activated microglia enabling antigen presentation to T cells potentially recruited to the eye^20, 21^. The RPE, choroid and retina of aged mice over-express several genes related to T-cells and their chemotaxis, antigen presentation, and adhesion^22, 23^. Collectively, these findings suggest a potential role of the adaptive immune system, in particular the T-lymphocytes, in the pathogenesis of AMD.

Activated CD4^+^ T cells can be differentiated based on production of signature cytokines into T helper (Th1), Th2, Th17 and regulatory (Treg) subsets^24^. The Th1 and Th17 subsets secrete pro-inflammatory cytokines and are also involved in neurodegenerative and neovascular processes^25–28^. The Th1 and Th17 subsets can be recognized and separated by their expression of the surface proteins CXCR3 and IL12RB2 or CCR6 and IL23R, respectively. To the best of our knowledge, the occurrence of these pro-inflammatory T cell subsets has not been investigated in AMD. The purpose of this study was to investigate any potential differences in the frequency of Th1 and Th17 cells in the systemic circulation of patients with different subgroups of AMD and controls. Moreover, we explored potential differences in the expression of the individual surface proteins, IL12RB2, IL23R, CCR6, and CXCR3. Finally, we assessed the co-expression of CXCR3 and CCR6 on CD4^+^ cells since we have previously found differences in the expression of these proteins in patients with multiple sclerosis^29^.

## Methods

This prospective case-control study was approved by the Regional Committee of Ethics in Research of the Region of Zealand (Journal No. SJ-142). Verbal and written consent was obtained from all participants before inclusion. We followed the ethical principles for medical research stated in the Declaration of Helsinki.

### Study participants, clinical diagnosis, and medical data

Participants were consecutively recruited from our Department of Ophthalmology at Zealand University Hospital, Roskilde, Denmark. We recruited patients with exudative and non-exudative AMD from our outpatient retinal program. Healthy relatives of the patients were invited to participate as age-matched control individuals. This was an intentional strategy to better match the control group. Since this was a hypothesis-driven study, our aim for the number of participants was based on our previous experience with studies of systemic lymphocytes in patients with AMD rather than a power calculation, which is at least 20 individuals in each group^9, 12, 30^. We thus recruited at least 20 individuals in each group and stopped including further participants after recruiting a total of 90.

Retinal diagnosis was made by trained ophthalmologists using bilateral ophthalmoscopic fundus examination using a 90 diopter lens in mydriasis, digital color fundus photography (Carl Zeiss, Jena, Germany), Spectral-Domain Optical Coherence Tomography and fundus autofluorescence imaging (Spectralis HRA-OCT, SLO Heidelberg Engineering, Heidelberg, Germany). Participants suspected of having exudative AMD were also subjected to fluorescein and indocyanine green angiography to confirm diagnosis. Participants were categorized into subgroups of AMD according to the Clinical Age-Related Maculopathy Staging (CARMS) system^31^. Participants were classified as having non-exudative AMD (equivalent to CARMS score 2–4) if they exhibited signs of pigment abnormalities associated with age-related maculopathy, approximately ≥10 small drusen or any intermediate drusen, drusenoid retinal pigment epithelial detachment or geographic atrophy. Participants were classified as having exudative AMD (equivalent to CARMS score 5) if they had signs of neovascular AMD, including nondrusenoid pigment epithelial detachments, serous retinal detachments, choroidal neovascular membrane with subretinal or sub-RPE hemorrhages, or subretinal fibrosis. Non-AMD controls (equivalent to CARMS score 1) had normal maculae without drusen or fewer than 10 small drusen without pigment abnormalities. Both eyes were examined, and if the CARMS stages differed between the two eyes, the higher CARMS stage (5 being the highest) was chosen.

All participants were subjected to a structured standardized interview where medical conditions and current medication use was recorded. Based on their smoking habits, participants were categorized as either never smokers (less than 100 cigarettes during lifetime), ex-smokers (more than 100 cigarettes during lifetime but not within the last 12 months), or current smokers (which included those who reported quitting within the last 12 months)^32^. Height and weight were measured and used to calculate the body mass index (BMI). The participants were categorized as being either physically active or inactive based on a single-sentence questionnaire validated for observational studies^4, 33^. Alcohol use was noted as units (=12 g ethanol) per week.

### Participant eligibility

Participants were not included if they reported being ill within the last five days, had a history of any form of ongoing cancer, or systemic inflammatory or autoimmune conditions. Participants were also not included if they were receiving immune-modulating therapy, had received anti-vascular endothelial growth factor (VEGF) with Ranibizumab (Lucentis®, Genentech, South San Francisco, CA, USA) or Aflibercept (Eylea®, Bayer, Leverkusen, Germany) within the last 30 or 60 days, respectively, or were affected by any eye condition that hampered confident grading of the patient’s maculae. No participant had received other forms of intravitreal therapy, e.g. bevacizumab or lampalizumab.

Venous blood was obtained in a 3.5 ml evacuated gel tube containing lithium-heparin to measure the systemic C-reactive protein concentration. Individuals with C-reactive protein concentrations higher than 15 mg/l (∼99th percentile of the general population) were excluded to avoid interference with undiagnosed infectious, inflammatory or malignant conditions^34^.

### Leukocyte preparation and flow cytometric analysis

Fresh blood (3 ml) from the antecubital vein was obtained from all participants in tubes containing ethylenediamine-tetraacetic acid coagulant, and preparation for flow cytometric analysis was begun within 4 hours. Blood sampling was performed prior to any fluorescein and indocyanine angiography to avoid interference^35^. The volume of blood used for each participant was equivalent to 5 × 10^5^ white blood cells and calculated using a white blood cell count in a hematology analyzer (Sysmex KX-21N^TM^, Sysmex Corporation, Kobe, Japan). The red blood cells were lysed by adding 10% red blood cell lysis buffer (Nordic Biosite AB, Täby, Sweden) to the entire blood sample for 10 minutes in the dark at room temperature. The cells were washed three times and after each wash centrifuged for 5 minutes at 500 G and finally resuspended in isotonic buffer (IsoFlow Sheath Fluid, Beckman Coulter, Inc, Brea, CA, USA). The following monoclonal anti-human antibodies were applied to the sample: Allophycocyanin-H7 CD4, Clone RPA-T4, IgG1 (catalog number 560158, BD Pharmingen, Franklin Lakes, NJ, USA); PE-CY7 CXCR3/CD183-PE-CY7 Clone 1C6 IgG1 (catalog number 560831, BD Pharmingen); Allophycocyanin IL12R-B2 Clone 305719 IgG1 (catalog number FAB1959A, R&D Systems, Inc, Minneapolis, MN, USA); FITC CCR6 Clone G034E3 IgG2B (catalog number 353412, Biolegend, San Diego, CA, USA); PE IL23R Clone 218213 IgG2B (catalog number FAB14001P, R&D Systems Inc). Corresponding fluorochrome-matched negative isotype controls were used in a separate tube and set at 1% (APC-H7 Mouse IgG1, k isotype control, catalog number 560167, BD Pharmingen; PE-Cy7 Mouse IgG1 k isotype control, catalog number 557872, BD Pharmingen; APC Mouse IgG1 isotype control, catalog number IC002A R&D Systems Inc; FITC Mouse IgG2b, k isotype control, catalog number 400310, Biolegend; Mouse IgG2b isotype control, catalog number IC0041P, R&D Systems Inc). After incubation at room temperature in the dark as recommended by the respective manufacturers, the cells were washed and resuspended in 300 µl of isotonic buffer. The stained cells (n = 100 000) were analyzed using BD FACSCANTO II flow cytometry (BD Biosciences, Franklin Lakes, NJ, USA) and Kaluza Software (v. 1.5.20365.16139, Beckman Coulter Inc., Pasadena, CA, USA).

**Figure 1:**
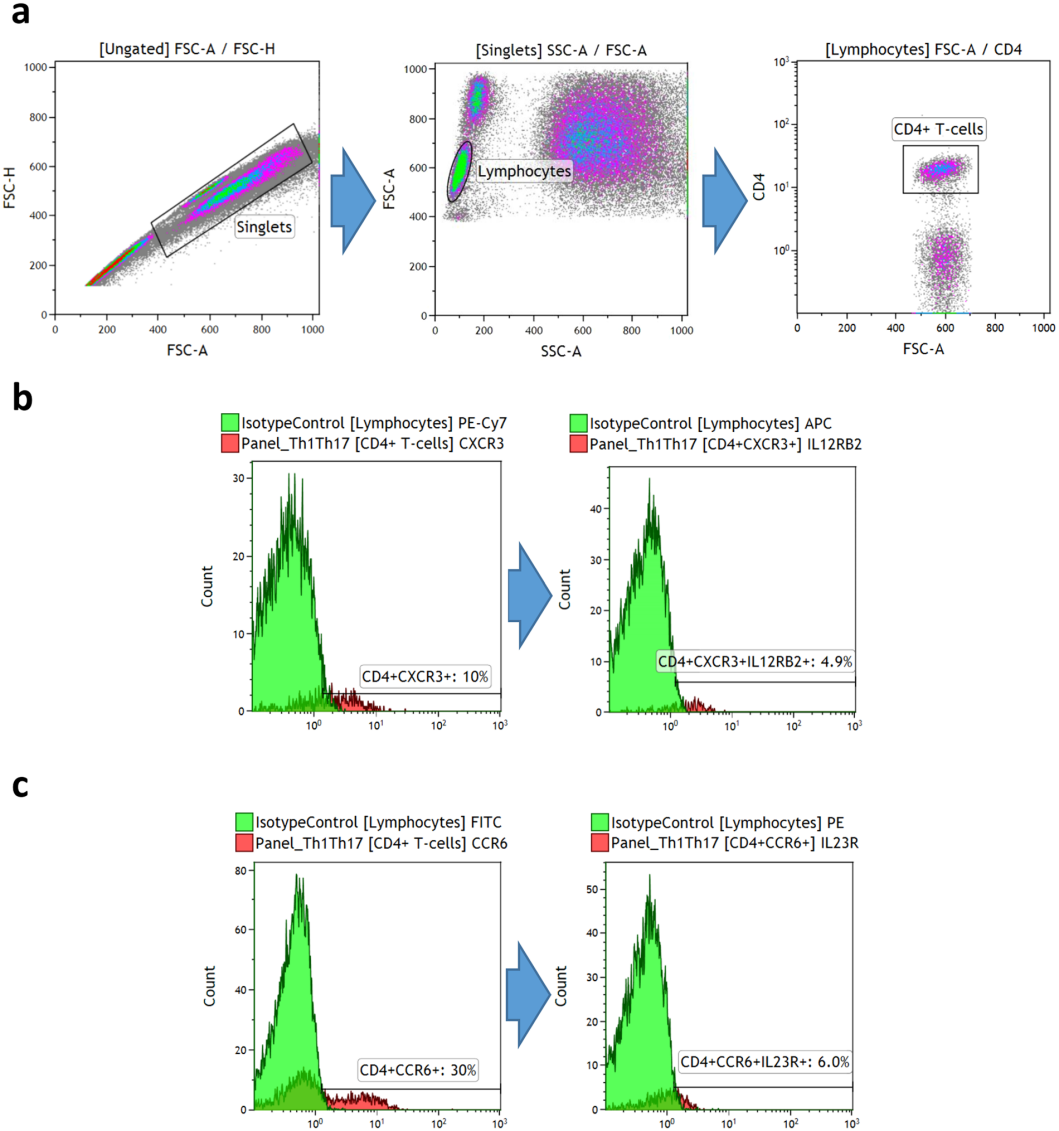
Gating strategies for identifying T helper 1 (Th1) and 17 (Th17) cells using Kaluza Software. (**a**) We first identified CD4^+^ T-cells by first identifying singlets (using forward height vs. area scatter), then lymphocytes (using forward vs. side scatter), and then the CD4^+^ lymphocyte population. (**b**) Th1 cells are CD4^+^CXCR3^+^IL12RB2^+^, so we first gated CD4^+^ and identified CXCR3^+^ cells, which we then gated to identify the IL2RB2^+^ cells. We used negative isotype control at 1% to distinguish positive populations from non-specific background signals. (**c**) Th17 cells are CD4^+^CCR6^+^IL23R^+^, so we first gated CD4^+^ and identified CCR6^+^ cells, which we then gated to identify the IL23R^+^ cells. We used negative isotype control at 1% to distinguish positive populations from non-specific background signals.

We plotted forward scatter area vs. height to identify singlets. Then, lymphocytes were gated using forward/side scatter plot. CD4^+^ cells were then isolated and the percentage of CD4^+^ cells expressing both CXCR3 and IL12RB2 (representing Th1 cells) and CCR6 and IL23R (representing Th17 cells) were assessed using two different dot-plot diagrams **(**Fig. 1 **)**. In order to look for potential differences in the individual surface proteins, we also examined the expression of IL12RB2, IL23R, CCR6, CXCR3. In addition, the co-expression of CXCR3 and CCR6 on CD4^+^ cells was quantified.

### Data analyses and statistics

First, demographic and clinical characteristics between groups were compared. We then explored differences between groups in the frequency of Th1 and Th17 cells, and the percentage of CD4^+^ T cells expressing the individual proteins. As aging introduces changes in the T-cell population, we performed correlation analyses between advancing age and frequencies of all CD4^+^ T cell subsets studied. Finally, we explored correlations between the CD4^+^ T cell subsets using group-specific correlation networks. We analyzed all data using SPSS 23 (IBM, Armonk, NY, USA). Data with normal distribution are presented using mean and standard deviation (SD), compared using the one-way analysis of variance (ANOVA) and the independent samples t-test, and correlated using Pearson’s correlation. As the median Th1/Th17 ratio and frequency of CD4^+^CXCR3^+^CCR6^+^ cells did not fit into normal distribution, these data are presented using median and interquartile range (IQR), compared using the Kruskal-Wallis’ one-way analysis of variance by ranks and the Mann-Whitney U test, and correlated using Spearman’s correlation. Categorical variables are presented in numbers and percentages, and compared using the χ^2^-test or Fisher’s Exact test when categories were <10. We consider a P-value below 0.05 as a sign of statistical significance.

## Results

### Participant characteristics

Of the 90 participants recruited in total, 32 were patients with exudative AMD, 26 were patients with non-exudative AMD (3 with CARMS 2, 9 with CARMS 3, and 14 with CARMS 4), and 32 were healthy control individuals. We excluded four participants (two patients with exudative AMD, one patient with non-exudative AMD, and one control individual) due to presumed ongoing acute immune response reflected by C-reactive protein concentrations higher than 15 mg/l. Another two participants, one control individual and one patient with non-exudative AMD, were excluded due to onset of severe allergic rhinitis and asthma exacerbation, respectively, on the day of recruitment. Participant characteristics of the 84 participants included for the analyses are summarized in Table 1. The groups did not differ significantly in demographic, lifestyle, and medical factors.

**Table 1:** Demographic, lifestyle, and medical characteristics of included study participants.

	Controls (n = 30)	Non-exudative AMD (n = 24)	Exudative AMD (n = 30)	P-value
Age, years, mean (SD)	72.5 (6.8)	76.2 (8.7)	76.9 (7.6)	0.066
Gender, n (%)				0.166
Female	17 (57)	19 (79)	17 (57)	
Male	13 (43)	5 (21)	13 (43)	
Smoking history, n (%)				0.513
Never	13 (43)	8 (33)	10 (35)	
Ex-smoker	11 (37)	10 (42)	16 (55)	
Current smoker	6 (20)	6 (25)	3 (10)	
BMI, kg · m^−2^, mean (SD)	26.7 (3.0)	25.9 (4.5)	24.6 (2.9)	0.054
Physically active, n (%)	15 (50)	11 (46)	13 (45)	0.916
Alcohol use, units/week, median (IQR)	3 (0 to 10)	2 (0 to 9)	3 (0 to 6)	0.828
Co-morbidities, n (%)				
Dyslipidemia	7 (23)	8 (33)	6 (20)	0.442
Hypertension	15 (50)	13 (54)	17 (57)	0.848
Other CVDs	7 (23)	8 (33)	6 (20)	0.540
C-reactive protein				0.166
<2.9 mg · L^−1^l, n (%)	20 (67)	18 (75)	18 (60)	
2.9–7.9 mg · L^−1^, n (%)	7 (23)	6 (25)	12 (40)	
8.0–14.9 mg · L^−1^, n (%)	3 (10)	0 (0)	0 (0)	

Abbreviations: n = number; AMD = age-related macular degeneration; SD = standard deviation; BMI = body mass index; CVDs = cardiovascular diseases. Missing data: smoking history is missing for one participant with exudative AMD, body mass index is missing for two participants with non-exudative AMD, and physical activity is missing for one participant with exudative AMD. P-values are calculated using analysis of variance (ANOVA) for age and body mass index; Kruskal-Wallis’ test for alcohol consumption, χ^2^-test for hypertension and physical activity; and Fisher’s Exact test for gender, smoking, dyslipidemia, other cardiovascular conditions, and C-reactive protein levels.

### Group differences in CD4^+^ T-cells’ surface protein expression and subtype

We quantified the percentage of CD4^+^ cells co-expressing CXCR3 and IL12RB2 to study the frequency of Th1 cells in blood (Table 2). Lower frequency of Th1 cells was observed among patients with exudative AMD (p = 0.049, ANOVA; p = 0.024, independent samples t-test between control and exudative AMD). We quantified the percentage of CD4^+^ cells co-expressing CCR6 and IL23R to study the frequency of Th17 cells in blood, and found no difference between the three groups (p = 0.351, ANOVA). The Th17/Th1 ratio was similar in the three groups (p = 0.973, Kruskal-Wallis’ test). No significant differences were found in the expression of IL12RB2, IL23R, or CCR6 on CD4^+^ T-cells. The expression of CD4^+^ T-cells expressing CXCR3 was significantly lower in patients with AMD (p = 0.025, ANOVA; p = 0.014, independent samples t-test between control and exudative AMD; p = 0.064, independent samples t-test between control and non-exudative AMD). The co-expression of CXCR3 and CCR6 on CD4^+^ T-cells was also lower in patients with AMD (p = 0.028, Kruskal Wallis’ test; p = 0.016, Mann-Whitney U-test between control and exudative AMD; p = 0.052, Mann-Whitney U-test between control and non-exudative AMD).

**Table 2:** CD4^+^ T-cell surface protein expression in patients with non-exudative age-related macular degeneration (AMD), patients with exudative AMD, and control individuals.

	Controls (n = 30)	Non-exudative AMD (n = 24)	Exudative AMD (n = 30)	P-value (group)	P-value (con. vs. non-ex. AMD)	P-value (con. vs. ex. AMD)
Th1 (CD4^+^CXCR3^+^IL12RB2^+^), mean (SD)	6.1 (4.8)	4.2 (3.3)	3.7 (2.2)	0.031^a^	0.098^b^	0.016^b^
Th17 (CD4^+^CCR6^+^IL23R^+^), mean (SD)	9.2 (5.8)	8.0 (7.1)	6.3 (5.5)	0.181^a^	—	—
Th17/Th1 ratio, median (IQR)	1.6 (1.0 to 2.2)	1.5 (0.9 to 2.5)	1.3 (0.7 to 2.7)	0.631^c^	—	—
CD4^+^IL12RB2^+^, mean (SD)	38.9 (8.4)	38.5 (8.8)	37.5 (5.3)	0.780^a^	—	—
CD4^+^IL23R^+^, mean (SD)	24.9 (13.4)	24.4 (15.3)	18.7 (13.1)	0.173^a^	—	—
CD4^+^CXCR3^+^, mean (SD)	13.9 (10.7)	9.4 (7.0)	8.2 (5.1)	0.019^a^	0.066^b^	0.012^b^
CD4^+^CCR6^+^, mean (SD)	28.8 (9.8)	21.1 (11.9)	22.9 (10.6)	0.024^a^	0.015^b^	0.030^b^
CD4^+^CXCR3^+^CCR6^+^, median (IQR)	4.1 (2.4 to 8.6)	1.6 (1.1 to 4.7)	2.2 (1.4 to 4.0)	0.009^c^	0.007^d^	0.014^d^

Abbreviations: n = number; AMD = age-related macular degeneration; Th1 = T helper 1; Th17 = T helper 17; SD = standard deviation. Groups are compared using: ^a^analysis of variance (ANOVA); ^b^independent samples t-test; ^c^Kruskal-Wallis test; ^d^Mann-Whitney U-test. Cell populations with significant differences are greyed out.

### Impact of aging

The range of age was similar between the three groups: 59 to 88 years in healthy controls, 58 to 96 in patients with non-exudative AMD, and 59 to 87 in patients with exudative AMD. In healthy controls, increasing age correlated with decreasing frequency of Th1 cells (ρ = −0.51, p = 0.005, Pearson’s correlation) and percentage of CD4^+^ T cells with co-expression of CXCR3^+^ and CCR6^+^ (ρ = −0.43, p = 0.022, Spearman’s correlation); and increasing age correlated with increasing Th17/Th1 ratio (ρ =  + 0.64, p = 0.022, Spearman’s correlation) and percentage of CD4^+^ T-cells expressing CXCR3(ρ =  + 0.45, p = 0.017, Pearson’s correlation) **(**Table 3 **)**. These correlations were absent in patients with non-exudative and exudative AMD, in which expression of none of the surface proteins studied correlated with age.

**Table 3:** Correlations between increasing age and CD4^+^ T-cells surface proteins are present in control individuals (CD4^+^CXCR3^+^, CD4^+^CXCR3^+^IL12RB2^+^, and CD4^+^CXCR3^+^CCR6^+^), but not in patients with non-exudative or exudative age-related macular degeneration (AMD).

	Controls (n = 30)		Non-exudative AMD (n = 24)		Exudative AMD (n = 30)	
	ρ	p-value	ρ	p-value	ρ	p-value
Th1 (CD4^+^CXCR3^+^IL12RB2^+^)^a^	−0.53	0.002	−0.20	0.343	−0.05	0.802
Th17 (CD4^+^CCR6^+^ IL23R^+^)^a^	+0.01	0.957	−0.05	0.810	+0.10	0.611
Th17/Th1 ratio^b^	+0.69	<0.001	−0.17	0.418	−0.11	0.572
CD4^+^IL12RB2^+a^	−0.15	0.420	−0.04	0.847	+0.21	0.256
CD4^+^IL23R^+a^	−0.04	0.817	−0.25	0.243	+0.15	0.439
CD4^+^CXCR3^+a^	−0.46	0.010	−0.17	0.426	−0.31	0.092
CD4^+^CCR6^+a^	+0.14	0.476	−0.08	0.727	−0.19	0.319
CD4^+^CXCR3^+^CCR6^+b^	−0.46	0.011	−0.25	0.246	−0.26	0.172

Abbreviations: n = number; AMD = age-related macular degeneration; Th1 = T helper 1; Th17 = T helper 17. Correlation coefficients (ρ) and p-values are calculated using: ^a^Pearson’s correlation; ^b^Spearman’s correlation. Cell populations with significant differences are greyed out.

### Group-specific correlation networks

**Figure 2:**
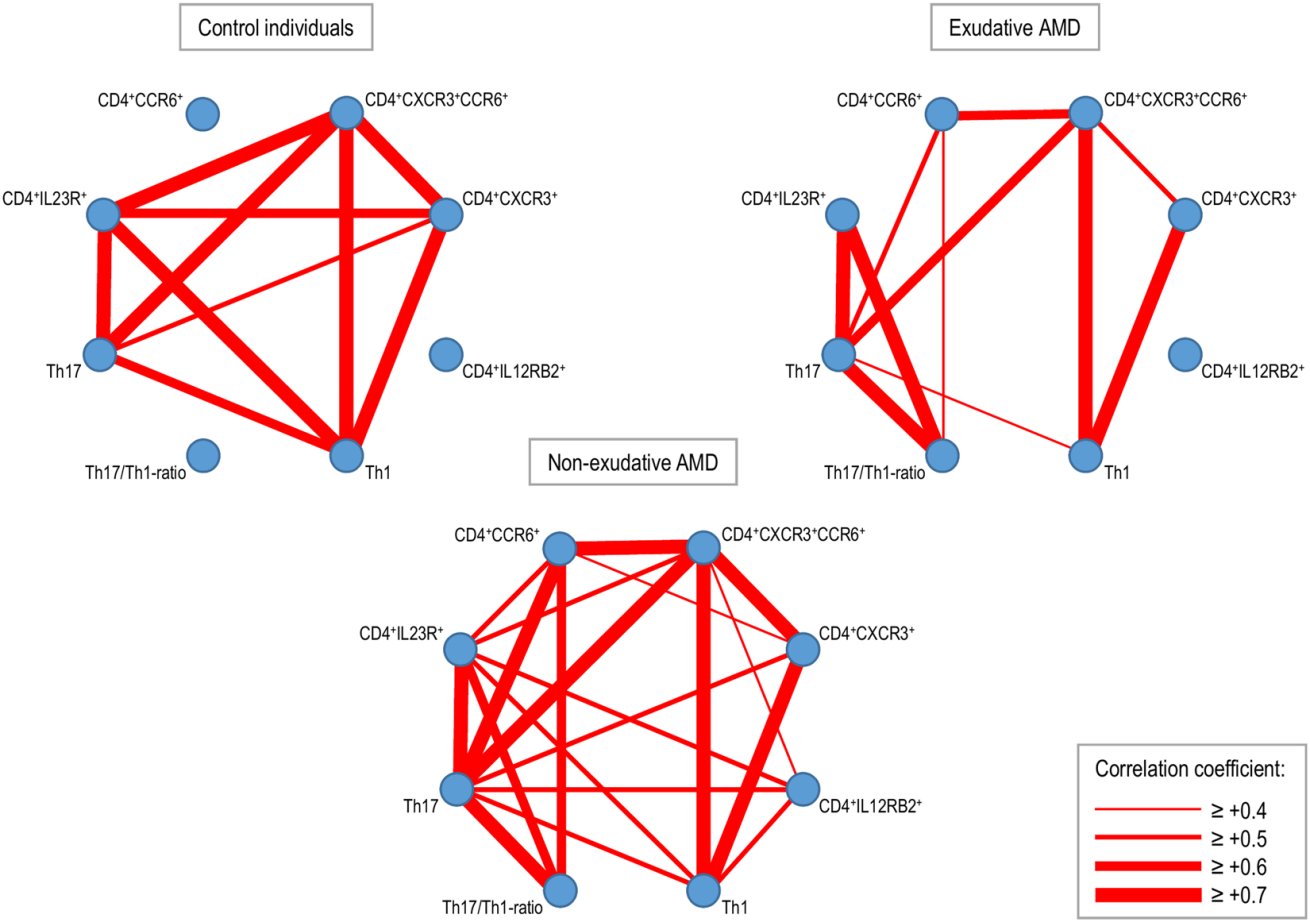
Correlation networks of CD4^+^ T-cell surface proteins in control individuals, patients with non-exudative age-related macular degeneration (AMD), and patients with exudative AMD. The relationships between surface proteins are calculated using Spearman’s correlation and the correlation coefficient is marked when ≥0.4 or ≤−0.4 using green or red lines, respectively. Differences in relationship patterns reveal that a complex systemic immune dysfunction may be present in patients with AMD.

The relationships between Th1, Th17, and expression of the individual cell surface proteins on CD4^+^ T-cells were investigated using group-specific correlation networks **(**Fig. 2 **)**. These networks revealed several differences in how the frequencies correlate with each other between the different groups, suggesting that a more complex systemic immune dysfunction in the adaptive immune system may be present in patients with AMD. Compared to control individuals, patients with exudative AMD lacked correlations of CD4^+^IL23R^+^ T-cells with Th1, CD4^+^CXCR3^+^, and CD4^+^CXCR3^+^CCR6^+^; and of CD4^+^CXCR3^+^ with Th17 cells. Instead, other correlations were present of CD4^+^CCR6^+^ with Th17 cells, Th17/Th1-ratio, and CD4^+^CXCR3^+^CCR6^+^ cells; and of Th17/Th1-ratio with Th17 and CD4^+^IL23R^+^ cells. Patients with non-exudative AMD had features of both control individuals and patients with exudative AMD, i.e. correlations of CD4^+^IL23R^+^ with Th1 and CD4^+^CXCR3^+^CCR6^+^ cells; of CD4+CCR6+ cells with Th17 cells, Th17/Th1-ratio, and CD4^+^CXCR3^+^CCR6^+^ cells; and of Th17/Th1-ratio with Th17 and CD4^+^IL23R^+^ cells.

## Discussion

The adaptive immune system is composed of highly specialized cells, T- and B-cells, designated to eliminate or prevent pathogen growth. T-helper cells are central players in adaptive immunity as they activate B-cells to secrete antibodies, macrophages to destroy ingested microbes, and cytotoxic T-cells to kill infected target cells^33^. T-helper cells, which differentiate from naïve CD4^+^ T-cells, may further differentiate into interferon-gamma (IFN-γ)-secreting Th1-cells (which support clearance of intracellular pathogens) under the influence of IFN-γ and interleukin (IL)-12. In a mouse model, ocular injection of carboxyethylpyrrole, a lipid peroxidation product associated with AMD in humans and AMD-like pathology in mice, resulted in a T-cell dependent adaptive immune response with Th1 differentiation, or IFN-γ production in general, seemingly playing an essential role in the process^36^.

We observed that patients with exudative AMD had lower frequency of CD4^+^CXCR3^+^ IL12RB2^+^ (Th1) cells compared to control individuals. Although generally considered to be pro-inflammatory, the exact role of Th1-secreted IFN-γ is unclear. It may be pro-inflammatory due to secretion of pro-inflammatory cytokines and chemokines which recruit monocytes/macrophages and T-cells, or anti-inflammatory due to upregulation of anti-inflammatory factors and inhibition of immune cells related to the autoimmune response^37^. The immune system and in particular the T-cell compartment undergoes age-related changes, a phenomenon known as immunosenescence. T-cells produce and secrete IL-2, a cytokine that induces cell proliferation and supports long-term growth of T-cells. As T-cells age, they lose their ability to produce and respond to IL-2 resulting in impaired T-cell function^38^. We have previously identified increased levels of aged, i.e. CD56^+^CD28^−^ T-cells in individuals with AMD^11^. In this study, we found that the frequency of Th1-cells decreased with advancing age in control individuals, but not in individuals with nonexudative or exudative AMD suggesting a dysfunctional aging of Th1 cells in patients with AMD. Accordingly, we found that the Th17/Th1 ratio increased with age in controls, but not in patients with AMD. Our findings in controls are supported by a previous study which showed that aging introduces a functional shift from Th1 to a Th2 response characterized by a reduction in the Th1 cytokine IFN-γ and an increase in the Th2 cytokine IL-4 and the immunoregulatory and B-cell promoting cytokine IL-10^39^.

An alternative differentiation from naïve CD4+ T-cells into Th17-cells is encouraged by TGF-β, IL-6 and to a lesser degree by TNF-α, IL-1β, and IL-23. Th17-cells are considered to be pro-inflammatory and have been implicated in autoimmune diseases and human inflammatory conditions, including multiple sclerosis^40^. IL-17 is a signature cytokine of Th17 cells, but is also secreted by γδ T-cells and innate lymphoid cells. IL-17 appears to play a role in AMD. Liu *et al*. reported that complement component C5a is increased in the circulation of AMD patients and that it promotes expression of the Th17 cytokines IL-17 and IL-22 by human CD4^+^ T-cells^41^. Also, an association between two single nucleotide polymorphisms in the IL-17 gene, rs2275913G/A and rs3748067C/T and AMD was reported^42^. IL-17 and TNF-α have been observed at higher concentrations in AMD patients and may be linked to a more favorable response to anti-VEGF therapy^43^. Altered DNA methylation of the IL17RC promoter has been observed in AMD patients leading to an elevated expression of its protein and messenger RNA in peripheral blood and affected parts of the retina and choroid^44^. In mice, IL-17 stimulates neovascularization in a VEGF-dependent manner after laser-injury^45^. In another study, modulating the IL-17 gene in *Ccl2* ^−/−^/*Cx3cr1* ^−/−^/*Crb1* ^rd8^ mice using an anti-inflammatory agent TSG-6 stabilized retinal lesions^46^. Taken together, these studies indicate that IL-17 may be involved in the pathogenesis of AMD by promoting retinal neovascularization. In our study, there was no absolute difference in the frequency of CCR6^+^ IL23R^+^ Th17 cells when comparing patients with exudative and non-exudative AMD with age-matched controls. In light of our findings, it is important to remember that apart from Th17 cells, γδ T-cells and innate lymphoid cells are important producers of IL-17. Indeed, Hasegawa *et al*. reported that γδ T-cells and THY1^+^ ILCs, but not Th17 cells were the relevant source of IL-17^45^. Thus, our findings suggest that the increase in IL-17 found by other groups in patients with AMD is not simply explained by a higher frequency of Th17-cells.

The chemokine receptor CXCR3 is a receptor for a range of chemoattractants (such as CXCL4, CXCL9, CXCL10, and CXCL11), facilitates entry of cells to sites of inflammation, and plays an important role in CXCL10-mediated inhibition of VEGF-induced angiogenesis^47, 48^. We have recently demonstrated a trend towards lower CXCR3 expression on circulating CD4^+^ CD69^+^ T cells (activated T-cells) in patients with exudative AMD compared to age-matched controls without AMD (p = 0.07)^12^. In this current study, we confirm the results from our previous study and report a lower expression of CXCR3 on CD4^+^ cells in patients with exudative AMD (p = 0.012) compared to controls without AMD. The frequency of CD4+IL12RB2+ did not differ between groups, and thus the reduction in CXCR3 expression on CD4+ cells may have been responsible for the observed difference in Th1 cells between our study groups. Interestingly, control individuals exhibit an age-related increase in CD4^+^CXCR3^+^, which does not occur in patients with AMD – in the exudative AMD group, there is even a small but insignificant trend (ρ = −0.31, p = 0.092) towards lower CD4^+^CXCR3^+^ with age. In addition, we found that the expression of CCR6 on CD4+ -cells is lower in patients with both non-exudative and exudative AMD compared to controls. The co-expression of CXCR3 and CCR6 on CD4^+^ T-cells was also lower in patients with any AMD, and the age-related decrease which we observed in controls did not occur in patients with AMD. T-cell subsets express CCR6, which is a receptor for the chemoattractant CCL20 (macrophage inflammatory protein 3α)^49, 50^. The chemoattractant activity is among T-cells specific to Th17 and Treg cells – Th17 cells both expresses the receptor and its ligand and the interaction is used to regulate the migration of Th17 cells to sites of inflammation, unlike e.g. Th1 and Th2 cells which lack a similar migratory response^50, 51^. Thus, it is unclear which role the systemic decrease in CCR6^+^ CD4^+^ T-cells and decreased co-expression of CXCR3 and CCR6 CD4^+^ T-cells plays, but this warrants further investigation.

When discussing isolated differences in systemic immunological alterations, it is important to remember that these cells are components of a large, complex, and interacting system. Thus, we also looked at relationships between the T-cell populations using correlation networks. Patients with AMD differed considerably from control individuals, particularly in correlations regarding CCR6^+^ CD4^+^ T-cells, CXCR3^+^ CD4^+^ T-cells, and the Th17/Th1-ratio. It is our understanding that two important messages can be derived from this. Firstly, this underscores that there may be a dysfunction in these immune cell populations. Future studies call for functional analyses of these subgroups. Secondly, the immune dysfunction in patients with AMD may be a part of a more complex picture of systemic immune dysfunction. When looking at the P-values in the comparisons in Table 2 and the correlations in Fig. 2, it seems that the group of non-exudative AMD may have trends towards immunological alterations similar to those in patients with exudative AMD albeit less pronounced. The results may also reflect disease heterogeneity. Clinical studies show that within 5 years 5 percent and within 15 years 15 percent of the patients progress from non-exudative AMD to exudative AMD^1^. Hence, a large number of patients with non-exudative AMD never develop the exudative type. This may explain, at least partly, why the immunological fingerprint of the non-exudative AMD illustrated as correlation networks looks as if the correlations in controls and exudative AMDs were put on top of each other. Future research must focus on further investigating potential immunological differences in subtypes of non-exudative AMD.

Some limitations of this study need to be considered. Firstly, the observational nature of our study design does not allow us to infer any causal relationship between Th1- and Th17-cell frequency and AMD. Thus, it is not possible to say whether the absence of the Th1 decrement in AMD is the cause of disease or an effect of disease-related medical or lifestyle factors. Since Th1 and Th17 cells cytokines are not specific to these cell types, it is difficult to exclude disparity between cells and their cytokines due to lack of functional analyses of cytokines, e.g. measurement of IFN-γ and IL-17 after stimulation. Moreover, since we did not measure frequency of other T cell subsets, such as Th2 and T regulatory cells, we cannot establish whether the changes observed in our study are restricted to specific T-cell subsets.

In conclusion, our study demonstrates a decreased frequency of CXCR3^+^IL12RB2^+^ Th1-cells in patients with AMD, but no such change in the frequency of CCR6^+^IL23R^+^ Th17-cells. The age-related decrement in Th1 frequency observed in healthy controls is absent in patients with AMD. The percentage of CD4^+^ T-cells expressing CCR6 was significantly lower in patients with non-exudative and exudative AMD. Moreover, we are able to reproduce previous findings, which show that exudative AMD is associated with a lower percentage of CD4^+^ T-cells expressing CXCR3 compared to controls. Our results suggest that patients with AMD exhibit alterations in the normal aging processes and in the complex interplay of the adaptive immune system. Taken together, our results support the notion that the adaptive immune system may be implicated in the pathogenesis of AMD and that AMD is a disease where normal aging processes become dysregulated.
